# Spinal Cord Stimulation and Related Health Information on Social Media: An Analysis of Instagram Posts

**DOI:** 10.7759/cureus.45129

**Published:** 2023-09-12

**Authors:** Serdar O Aydin, Omer Tasargol

**Affiliations:** 1 Neurosurgery, Kartal Dr. Lutfi Kirdar City Hospital, Istanbul, TUR; 2 Anesthesiology, Dr. Burhan Nalbantoglu State Hospital, Nicosia, CYP

**Keywords:** internet, instagram, health communications, content analysis, chronic pain management, social media platform, digital health awareness, online platform, spinal cord stimulation

## Abstract

Introduction: Spinal cord stimulation (SCS) has been a well-established, effective, minimally invasive procedure for the treatment of chronic medically refractory neuropathic pain involving the limbs and trunk. Social media platforms, including Instagram, are increasingly being used for medical education and sharing patient experiences. This study aimed to investigate posts related to SCS on Instagram.

Methods and materials: This study presents a comprehensive analysis of Instagram posts utilizing the hashtags #spinalcordneuromodulation, #spinalcordstimulation, #spinalcordstimulationsurgery, #spinalcordstimulationtherapy, and #spinalcordstimulationimplant, all of which were collected on August 1, 2023. The outcomes were organized into four distinct source-based categories: posts generated by medical practitioners (both surgeons and non-surgeons); content shared by medical organizations; content created by patients; and content from indeterminate categories. The content was further classified based on its nature, encompassing educational material and reflections on patient or physician experiences. Moreover, the impact of users was evaluated in terms of their follower count and the count of posts.

Results: The search yielded a total of 4983 posts. The majority of posts were created by medical practitioners (38.53%, n = 1920). The distribution of remaining Instagram posts about SCS was as follows: medical organizations for 35% (n = 1744), patients for 24.12% (n = 1202), and indeterminate categories for 2.35% (n = 117). Among the collected posts, 348 (41.4%) originated from accounts associated with medical practitioners, 286 (34%) from medical organizations, 145 (17.2%) from patients, and 62 (7.4%) remained unspecified. The statistical analysis revealed a significant difference in follower distribution between medical practitioners and all other groups (p<0.001). Reported side effects included pain over the implant (n = 257; 88.92%), lead migration (n = 18; 6.22%), infection (n = 9; 3; 11%), and seroma (n = 5; 1.73%).

Conclusions: When searching for posts about SCS on Instagram, one is more likely to encounter posts authored by medical practitioners that are mostly focused on educational content. Posts created by medical practitioners may be overshadowed and buried among numerous other posts created by patients. We suggest posting educational medical content with the hashtag #MedEd in an attempt to make educational content more easily accessible.

## Introduction

Spinal cord stimulation (SCS) represents a burgeoning, minimally invasive treatment approach for managing chronic medically refractory neuropathic pain involving the limbs and trunk. In the context of SCS, electrical impulses are administered directly to the spinal cord, aiming to suppress the relay of nociceptive signals to the cerebral cortex [1]. Annually, around 50,000 individuals worldwide undergo SCS as a therapeutic option for conditions including post-laminectomy syndrome, diabetic neuralgia, or complex regional pain disorders, and the outcomes vary widely among patients [2]. As the predominant form of neuromodulation, its adoption has escalated over recent years and is endorsed by guidelines both in European countries and the United States [3,4]. Spinal cord stimulation enables tailored pain control and management on a personal patient basis, necessitating active patient collaboration for optimization [5].

The Internet has revolutionized the dissemination of medical knowledge, becoming a crucial source for health information by quickly sorting through vast amounts of data. With a user base surpassing a billion, Instagram, an image-centric social media platform, dominates the digital sphere [6]. The widespread presence of Instagram has transformed it into an educational resource for patients and a visual guide for medical professionals seeking knowledge. Moreover, given the intrinsic power of visuals in data sharing and communication, platforms such as Instagram could be strategically valuable for health-centric dialogues and discussions. Previous studies affirm that content augmented with visual aids not only garners heightened retention but also extends recall longevity and accuracy in contrast to text-exclusive messages [7]. 

In the realm of interventional pain medicine practice, the provision of clear visual guidance holds important significance in defining relationships among anatomical structures, clinical proficiencies, and medical knowledge. Many medical institutions routinely create comprehensive educational multimedia resources tailored for advanced invasive interventions, such as the SCS procedure. Recently, the Internet has emerged as a valuable source of medical information for physicians. However, a current concern persists that medical discussions on the Internet might be disseminated in a manner tainted by inaccuracies, a lack of structure, or the absence of filtering mechanisms [8-11]. This emphasizes the need for implementing strategies aimed at evaluating and overseeing the content of online platforms like Instagram, ensuring precision and dependability.

The primary aim of this study was to assess the authenticity, reliability, and comprehensiveness of Instagram posts encountered by patients and medical professionals seeking information about SCS on Instagram. The secondary objective was to assess the attributes and sources of Instagram posts related to SCS.

## Materials and methods

Study design and ethical consideration

This study presents a non-interventional, descriptive analysis of online search behavior, excluding engagement with human subjects or animals. Due to the public accessibility of the assessed data, the ethics committee's approval was waived.

Data collection

On August 1, 2023, the hashtags #spinal cord neuromodulation, #spinal cord stimulation, #spinal cord stimulation surgery, #spinal cord stimulation therapy, and #spinal cord stimulation implant were explored utilizing the Instagram tool. The data collected on that date were accurate, and only posts in the English language were subjected to assessment. Posts that were duplicates or inaccessible were excluded from the analysis. The gathered posts were categorized into four distinct groups: medical practitioners (encompassing orthopedic surgeons and neurosurgeons, as well as non-surgical specialists such as interventional radiologists, practitioners in physical medicine and rehabilitation, anesthesiologists, and pain physicians); patients; medical organizations; or indeterminate categories. The content was further stratified based on typology, either educational or patient or physician experiences. Additionally, the impact of users was considered, including the count of posts and followers. Both captions and associated hashtags were meticulously reviewed and statistically analyzed. 

Statistical analysis

Statistical analysis was conducted using SPSS Statistics, version 24.0 (IBM Corp., Armonk, NY, USA). The normality of quantitative variables was assessed through the Shapiro-Wilk test. Descriptive statistics were employed to present the data, including counts (n), percentages (%), the mean, and the standard deviation. In instances where the overall test yielded significance, a subsequent Wilcoxon signed-rank test was conducted for pairwise comparisons. Statistical significance was defined as p<0.05.

## Results

**Figure 1:**
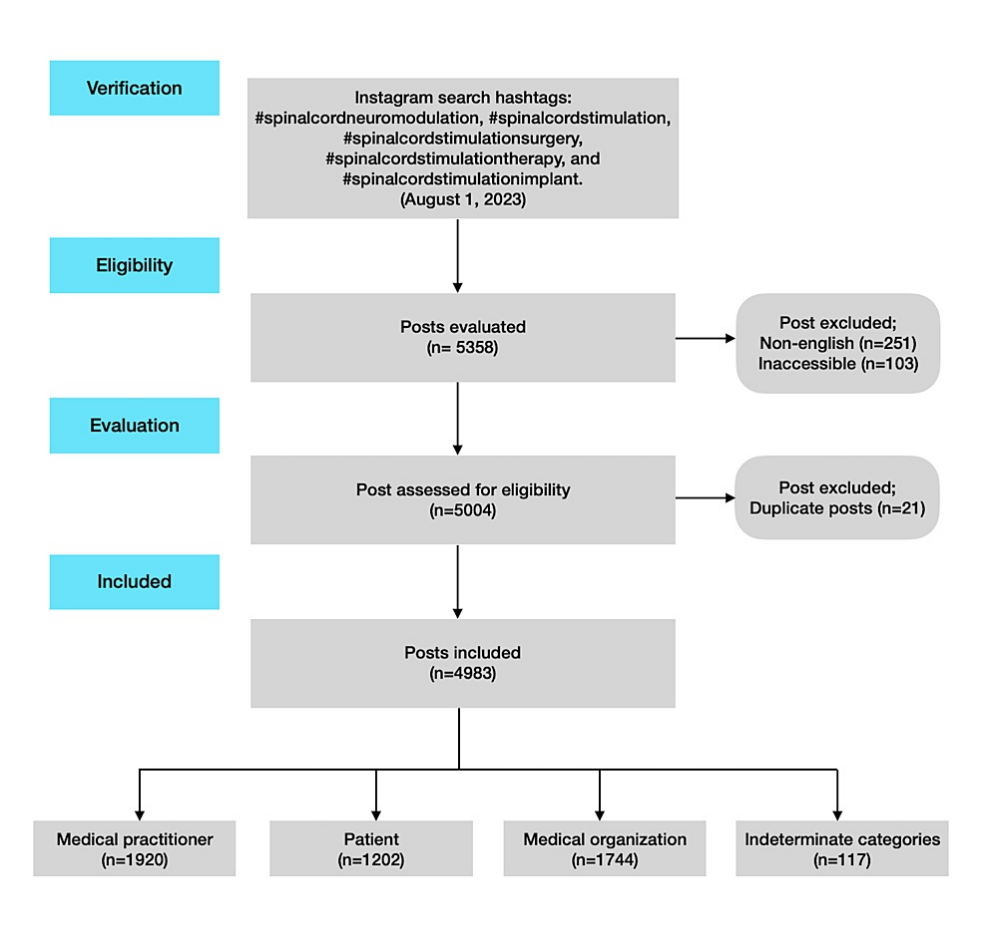
Schematization of how Instagram posts were selected

A total of 4983 Instagram posts underwent evaluation. The process of selection for the posts is illustrated in Figure 1. There were 841 distinct accounts categorized as medical practitioners, patients, medical institutions, or indeterminate categories. These accounts were distributed as follows: 41.4% were medical practitioners (n = 348), 17.2% were patients (n = 145), 34% were medical organizations (n = 286), and 7.4% were deemed indeterminate categories (n = 62) (Table 1).

**Table 1:** Characteristics of the selected Instagram posts

User	N (%)	No. of followers (mean±SD)
Medical practitioner	348 (41.4%)	1567,856 (4505.33±18656.22)
Surgeon (orthopedic or neurosurgical)	196 (56.3%)	946,723 (4830.21±9232.56)
Non-surgeon (interventional radiology, physical medicine and rehabilitation, anesthesiology, and pain management specialists)	152 (43.7%)	621,133 (4086.40±7982.25)
Patients	145 (17.2%)	45,258 (312.12±4121.36)
Medical organizations	286 (34%)	184,122 (643.78±9565.31)
Indeterminate categories	62 (7.4%)	15,628 (252.06±1086.43)

Most of the posts were created by medical practitioners (38.53%, n = 1920). The distribution of the remaining Instagram posts about SCS was as follows: medical organizations at 35% (n = 1744), patients at 24.12% (n = 1202), and indeterminate categories at 2.35% (n = 117). Among the 145 patient accounts, 99% were experience-based, while 1% were education-focused. In contrast, posts authored by medical practitioners were educational 86% of the time and constituted personal experiences 14% of the time. Hospital-related posts were predominantly educational (95%), contrasting with the 5% representing experiential content (Table 2).

**Table 2:** Analysis of the content in the selected Instagram posts SD: standard deviation

User	Total no. of posts (mean±SD)	Educational	Experiential
Medical practitioner	1920 (5.51±3.21)	86%	14%
Surgeon	941 (4.8±)	90%	10%
Non-surgeon	1158 (7.61±)	82%	18%
Patient	1202 (8.28±14.32)	1%	99%
Medical organizations	1744 (6.09±4.21)	95%	5%
Indeterminate categories	117 (1.88±7.64)	-	-

The examination focused on the diversity in follower count distribution among the four distinct groups. Pairwise comparisons unveiled notable differences in the distribution of posts between medical practitioners and patients (p 0.001), medical organizations (p<0.001), and indeterminate categories (p<0.001). Furthermore, the analysis established a statistically significant distinction in the count of posts between medical practitioners and patients (p<0.001) and indeterminate categories (p<0.001) (Table 3).

**Table 3:** Difference in the number of posts and followers * p<0.05 is considered statistically significant ^a ^Wilcoxon signed-rank test was performed for pairwise comparison

User groups	No. of posts (p-value)^a^	No. of followers (p-value)^a^
Medical practitioner vs. patient	<0.001*	<0.001*
Medical practitioner vs. medical organization	0.061	<0.001*
Medical practitioner vs. indeterminate categories	<0.001*	<0.001*
Patient vs. medical organization	0.073	0.616
Patient vs. indeterminate categories	<0.001*	0.870
Medical organization vs. indeterminate categories	<0.001*	0.747

**Figure 2:**
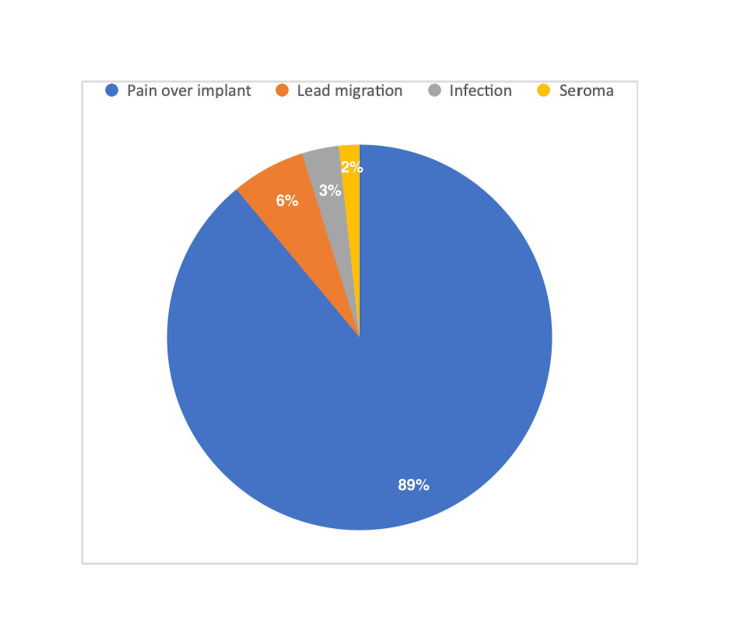
The distribution of reported side effects

The posts were examined to identify potential side effects. Patient accounts were categorized based on expressed side effects, including pain over the implant, lead migration, infection, bleeding, device migration, and seroma. In the analyzed posts, content referenced side effects. These reported side effects were distributed as follows: pain over the implant (n = 257; 88.92%), lead migration (n = 18; 6.22%), infection (n = 9; 3.11%), and seroma (n = 5; 1.73%) (Figure 2).

## Discussion

In this study, we evaluated the content of SCS on the rapidly emerging digital platform, Instagram. Our findings revealed that accounts managed by medical professionals and medical organizations tend to dominate the platform in terms of popularity. Despite the widespread presence of medical professionals and organizations on Instagram, 24% of the posts related to SCS were created by patients. Posts created by medical professionals were mostly educational.

Chronic pain imposes a significant burden on society, both in terms of economic implications and social consequences. The rates of spinal surgery, particularly spinal fusion, have experienced a rapid and substantial rise in the last decade [12,13]. The opioid epidemic in the United States emerged as one approach to pain management; however, it has brought about numerous adverse effects with catastrophic implications [14,15]. From a clinical perspective, SCS has been employed safely and effectively for over 20 years in the management of spine-related chronic pain syndromes [16]. Recent advancements in this domain have ushered in novel theories and approaches, redefining the utilization of SCS in the precision-driven and efficacious management of various chronic pain syndromes such as refractory angina pectoris, peripheral vascular disease, and complex regional pain syndrome, while mitigating the common adverse effects associated with alternative methods of chronic pain treatment such as opioids, injections, and radiofrequency procedures [17,18].

Recently, e-learning methodologies encompassing video and image-based formats have emerged as integral components of education [19,20]. Previous studies showed the advantages of incorporating visual elements into learning, particularly medical education [21]. Instagram, by virtue of its imagery-centric structure, stands as a well-suited platform for the wide-ranging dissemination of interventional medical education. Our findings showed that the majority of physicians' posts provide educational content. The use of hashtags in social media posts engenders a situation where educational materials might become overshadowed and obscured within the multitude of other posts. To enhance accessibility, we propose the inclusion of relevant hashtags like #MedEd or #MedEdu alongside educational medical content. 

Spinal cord stimulation is a complex pain treatment method involving an implanted device. Understanding its potential benefits and intricacies can be challenging for both patients and clinicians, as it utilizes low-level electrical signals to alleviate pain directly in the spinal cord. Besides, questions about whether spinal cord stimulators are MRI compatible, how to drive with one, and the removal process or trial period should be explained in detail to patients. Typically, patients may request to gather insights about SCS from their medical practitioners. However, research reports that it is a matter of concern that the informed consent process, a pivotal element in interventional practice, frequently remains inadequately fulfilled within surgical procedures. As a result, a considerable 74% of internet users engage in the quest for medical knowledge [22]. Here, Instagram emerges as one of the foremost platforms for accessing such information.

Instagram, a prominent visual-centric social media networking platform, is a global phenomenon with a staggering membership exceeding one billion individuals. Within the dynamic ecosystem of Instagram, users have the capability to share a diverse array of multimedia content, encompassing images and videos, through a variety of formats, including single posts, reels, stories, and Instagram TV. Moreover, Instagram facilitates global connectivity for users, enabling interactions spanning continents through mechanisms such as likes, comments, direct messages, and hashtags. Although Instagram's inception was not centered around the propagation of medical knowledge or healthcare education, the transformative influence of the Internet revolution has profoundly affected healthcare platforms. Consequently, it has emerged as one of the foremost repositories of health-related data, underscoring its pivotal role as a widely embraced source of medical insights.

The result of this present study showed that 24% of posts emanated from individual accounts, detailing personal experiences. Similarly, previous studies examining the impact of social media on health information found that the posts were mostly generated by patients and involved the sharing of personal experiences [23,24]. Generally, patient experiences hold utility for prospective patients seeking insights into SCS. Patients leverage the internet not only for the purpose of gathering health-related information but also for actively disseminating medical knowledge. Among the analyzed posts, pain over the implant, lead migration, infection, and seroma were the most reported side effects. The reporting of these side effects may adversely affect other patients because of the frequency of their mention. It's important to note that these information-sharing activities are not limited to patients alone; their family members also play a role in this process. However, it is imperative to acknowledge the potential bias inherent in the viewpoints of patients who have undergone SCS. This inherent bias could inadvertently influence prospective patients, leading to the dissemination of unfiltered and often inaccurate information. The lack of contributions authored by medical professionals or organizations underscores the missed potential of Instagram as a channel for the dissemination of dependable information regarding SCS. Physicians and medical societies have a significant responsibility for presenting information concerning SCS. Disseminating precise and dependable medical information via social media platforms plays a pivotal role in equipping patients with the requisite knowledge to make informed decisions concerning their healthcare. Although professionalism and ethics have been integral to medicine, the advent of social media necessitates unique considerations. Existing online guidelines from medical societies have typically been directed at specific healthcare practitioners, resulting in a lack of comprehensive guidance for interventional pain medicine physicians navigating the digital realm. Another notable deficiency in this regard is the lack of standardization for research on health-related topics conducted on social media platforms like Instagram [25-27]. Methodological adjustments in this regard would not only facilitate the planning of future studies but also enhance the value of the literature.

The posts created by physicians or medical organizations were mostly educational. In the realm of online health information dissemination, it is advisable that content creation and publication be entrusted exclusively to reputable medical professional societies and qualified specialists. This approach serves to mitigate the proliferation of unregulated, substandard, or unsuitable information that could potentially mislead or harm individuals seeking medical insights. On the other hand, it is important to note that patients' grasp of medical information can be influenced by both their level of health literacy and the intricacy of the employed terminology. While it may appear contradictory, following established standards and having skilled authors explain complex ideas concisely can make reliable medical information more accessible to the public.

This study has several limitations that warrant consideration. Primarily, the assessment was confined to a single day, potentially overlooking the evolving discussions intrinsic to Instagram's dynamic nature. Moreover, the exploration of alternative keywords associated with SCS could have enriched the search process. Notably, the absence of demographic data regarding users stands out as a notable shortcoming, attributed to Instagram's unavailability of these variables for analysis. Furthermore, this study's exclusive focus on English posts represents another constraint, and posts were not classified as video or photo. Lastly, acknowledging that patients may explore information on platforms beyond Instagram, there's a pertinent need for further research that assesses and contrasts diverse platforms. It is important to note that the findings cannot be generalized to encompass other social media platforms, given that Instagram was the exclusive platform subjected to analysis. 

## Conclusions

The findings revealed that accounts by physicians were the most popular and the most focused on sharing educational content. Posts created by physicians are more likely to be encountered when searching for information on SCS; however, these posts may become overshadowed and obscured within the multitude of other posts. It is important to encourage reliable Instagram accounts established by reputable pain management organizations to create more online information. Using hashtags such as #MedEd or #MedEdu alongside educational medical content may improve accessibility. Further studies are warranted to investigate the content provided by Instagram as a tool for health communication and medical education in the field of interventional procedures.
